# Changes in the 24-h movement behaviors during the transition to retirement: compositional data analysis

**DOI:** 10.1186/s12966-022-01364-3

**Published:** 2022-09-15

**Authors:** Kristin Suorsa, Tuija Leskinen, Jesse Pasanen, Anna Pulakka, Saana Myllyntausta, Jaana Pentti, Sebastien Chastin, Jussi Vahtera, Sari Stenholm

**Affiliations:** 1 Department of Public Health, University of Turku and Turku University Hospital, Turku, Finland; 2 Centre for Population Health Research, University of Turku and Turku University Hospital, Turku, Finland; 3 Center for Life Course Health Research, Faculty of Medicine, University of Oulu, Oulu, Finland; 4 The Population Health Unit, Finnish Institute for Health and Welfare, Helsinki, Finland; 5 Department of Psychology and Speech-Language Pathology, University of Turku, Turku, Finland; 6 Clinicum, Faculty of Medicine, University of Helsinki, Helsinki, Finland; 7 School of Health and Life Science, Glasgow Caledonian University, Glasgow, Scotland; 8 Department of Movement and Sports Sciences, Ghent University, Ghent, Belgium

**Keywords:** Accelerometer, Occupation, Sleep, Sedentary time, Physical activity, Compositional data analysis

## Abstract

**Background:**

Transition to retirement is shown to affect sleep, sedentary time and physical activity, but no previous studies have examined how retirement changes the distribution of time spent daily in these movement behaviors. The aim of this study was to examine longitudinally how the composition of 24-h movement behaviors changes during the transition to retirement using compositional data analysis (CoDA).

**Methods:**

We included 551 retiring public sector workers (mean age 63.2 years, standard deviation 1.1) from the Finnish Retirement and Aging study. The study participants wore a wrist-worn ActiGraph accelerometer for one week 24 h per day before and after retirement, with one year between the measurements. The daily proportions to time spent sleeping, in sedentary behavior (SED), light physical activity (LPA) and moderate-to-vigorous physical activity (MVPA) were estimated using the GGIR package. Changes in the daily proportions of movement behaviors were examined using Compositional Data Analysis version of linear mixed models.

**Results:**

In general, the proportion of time spent in active behaviors decreased relative to time spent in passive behaviors after retirement (*p* < .001). This change depended on occupation (occupation*time interaction *p* < .001). After retirement manual workers increased the proportions of both sleep and SED in relation to active behaviors, whereas non-manual workers increased the proportion of sleep in relation to active behaviors and SED. The proportion of MVPA decreased relatively more than the proportion of LPA (*p* = 0.01), independently of gender and occupation.

**Conclusions:**

Retirement induced a decrease in the proportion of time spent in active behaviors, especially time spent in MVPA. Future studies are needed to find ways to maintain or increase daily physical activity levels at the cost of sedentary behaviors among retirees.

**Supplementary Information:**

The online version contains supplementary material available at 10.1186/s12966-022-01364-3.

## Introduction

Retirement is an important life transition that affects sleep, sedentary time (SED) and physical activity, which are most often referred as “movement behaviors” in time-use epidemiology [1, 2]. Retiring from work has been found to associate with increased sleep duration [3, 4] and increased sedentary time (SED) measured with wrist-accelerometry, especially among women retiring from manual occupations [5, 6]. Moreover, pre-retirement occupation seems to explain changes in daily physical activity after retirement as women retiring from manual occupations were observed to become less physically active, whereas men from non-manual occupations were shown to increase their physical activity slightly after retirement [7]. However, in these previous studies, daily behaviors have been studied in isolation from each other, and thus, it is not known how 24-h movement behaviors change in relation to each other after the transition to retirement.

Examining sleep time, SED and physical activity separately from each other ignores the fact that changing time-use in one component also leads to changes in at least one of the remaining components, as they all are components of the 24-h day. With respect to previous findings on changes in the single movement behavior components during the transition to retirement, it is also important to examine how behaviors change in relation to each other. For example, increasing sleep duration to the recommended level is a positive change for health, but it may be more beneficial if increased sleep time reduces SED rather than physical activity [8]. On the other hand, increasing sleep or sedentary time in relation to light physical activity (LPA) versus moderate-to-vigorous physical activity (MVPA) may result in different health outcomes [8, 9].

The aim of this study was to examine how composition of 24-h movement behaviors changes during the transition to retirement using compositional data analysis (CoDA). CoDA enables examining the co-dependent movement behaviors that are relative components of a fixed amount of time, such as the 24-h movement behaviors [10, 11]. Since previous studies have shown that changes in sedentary time and physical activity strongly depend on gender and occupation [5–7], we also conducted analyses by these determinants. To the best of our knowledge, no previous studies have examined how retirement changes distribution of time spent daily in all movement behaviors.

## Methods

### Study population

This study is based on the Finnish Retirement and Aging Study (FIREA) which is an ongoing longitudinal cohort study of older adults in Finland established in 2013 [12]. The eligible population for the FIREA study cohort included all public sector employees whose individual retirement date was between years 2014 and 2019 and who were working in year 2012 in one of the 27 municipalities in Southwest Finland or in the 9 selected cities or 5 hospital districts around Finland. Information on individual retirement date was obtained from Keva Public Sector Pensions, the pension insurance institute for the public sector in Finland. The FIREA study cohort members were first contacted 18 months prior to their estimated retirement date by sending them a questionnaire inquiring for example about work factors, health behaviors, and health and functioning. Thereafter the FIREA cohort members were followed with annual questionnaires.

The FIREA study is conducted in line with the Declaration of Helsinki, and has been approved by the Ethics Committee of Hospital District of Southwest Finland. The participants provided written informed consent before participation.

The eligible study population for the present FIREA activity sub-study included those Finnish-speaking FIREA cohort members who completed the first questionnaire while they were still working and whose estimated statutory retirement date was between years 2016 and 2019 (n = 2663). These participants were contacted by mail to invite them to take part in the activity sub-study and of them 908 (34% of the eligible) agreed and returned the written informed consent. There were slightly more women and non-manual workers, and fewer self-reported inactive people among the participants who consented to the FIREA activity sub-study when compared with those who were eligible, but did not consent [5, 13].

The activity sub-study participants were followed up annually with accelerometer measurements across the retirement transition. To determine the timing of retirement, the actual retirement day was inquired during each phase of the data collection. For the present study we included 563 participants who had successfully used accelerometer before and after the transition to full-time statutory retirement, with one year in between the measurements (rest of the participants were not yet retired), and who were not on sick leave during the measurement week before retirement and did not have an acute condition limiting their mobility (for instance an injury) during the measurement week before or after retirement.

### Accelerometer measurements

Triaxial ActiGraph wActiSleep-BT and wGT3X-BT accelerometers (ActiGraph, Pensacola, Florida, US) were used to estimate the 24-h movement behaviors, that is sleep, SED, LPA and MVPA before and after retirement. Accelerometers were initialized to record at 80 Hz sampling frequency and mailed to the participants. Participants were asked to wear the device on their non-dominant wrist for seven consecutive days and nights at all times, including water-based activities. Participants were also provided a daily log, where they were asked to record the date, waking time, bedtime and working times on each measurement day. Data from the accelerometers were downloaded in the ActiLife software, version 6.13 (ActiGraph, Pensacola, Florida, US).

The R-package GGIR version 1.7–1 was used to analyze raw acceleration data from the wrist-worn accelerometers in the R statistical software, version 3.5.1 (R Foundation for Statistical Computing, Vienna, Austria, https://cran.r-project.org/). Accelerometer data were processed using the principles in the GGIR [14–16]. We included wear time between the first and last recorded times in the participant log and excluded non-wear time using the algorithm in the GGIR package. The algorithm classified non-wear time using 15 min time blocks based on the characteristics of the 60 min time window centered at these 15 min. A block was classified as non-wear time if the standard deviation of the 60 min window was less than 13.0 m*g* (milli gravity-based acceleration unit, where 1 *g* = 9.81 m/s^2^) for at least two out of the three axes or if the value range, for at least two out of three axes, was less than 50 m*g* [15, 17]*.* Sleep time was detected based on the combination of the daily logs and algorithm of the GGIR package [18], so that sleep was defined as periods of time within the bedtime and waking times reported in the daily logs during which there was no change larger than 5° in the arm angle over at least 5 min. Wake time SED, LPA and MVPA were defined using the threshold values of < 30 m*g*, at least 30 m*g* but less than 100.6 m*g,* and ≥ 100.6 m*g*, respectively [19, 20].

The measurement day was determined from each measurement day’s bedtime to the next measurement day’s bedtime. The analyses were restricted to valid measurement days with at least 10 h of waking wear time. No specific restrictions were made regarding night duration. The average duration of each valid measurement day was 23.8 h (range 20.6 − 27.7, interquartile range (IQR) 23.5 − 24.0) before and 23.8 h (range 20.6 − 27.1, IQR 23.6 − 24.0) after retirement, indicating very good compliance for the 24-h measurement. Time spent in each behavior were converted to proportion of the 24-h day [2].

Those participants who had less than four valid measurement days in either or both of the measurement points were excluded (*n* = 7), leaving 556 participants to the analytical sample. The mean number of valid measurement days was 6.9 (range 4 − 8) per measurement week before retirement and 6.9 (range 4 − 7) after retirement. The mean number of accelerometer-detected nights was 6.0 (range 4 − 7) before retirement and 6.0 (3 − 7) per measurement week after retirement.

### Assessment of pre-retirement characteristics

Gender, date of birth, and occupational title were obtained from the Keva Public Sector Pensions register. Participants were divided into two groups according to the occupational titles of the last known occupation preceding retirement by using the International Standard Classification of Occupations (ISCO) [21]: manual workers (for instance practical nurses, cooks, cleaners, maintenance workers; ISCO classes 5–9) and non-manual workers (for instance teachers, physicians, registered nurses, technicians; ISCO classes 1–4).

Other participant characteristics were obtained from the questionnaire preceding the transition to retirement. Smoking was categorized as former/non-smokers and current smokers. Participants were asked about doctor-diagnosed chronic diseases (angina pectoris, myocardial infarction, cerebrovascular disease, claudication, osteoarthritis, osteoporosis, sciatica, fibromyalgia, rheumatoid arthritis and diabetes), which were categorized as no vs. yes (one or more). Self-reported mobility limitation was assessed with a question about difficulties in walking two kilometers and categorized as no vs. yes (somewhat or markedly difficult) [22, 23]. Body mass index (BMI) was calculated from self-reported weight and height (kg/m^2^).

### Statistical analyses

Descriptive information on participant characteristics is presented using means and standard deviations for continuous variables and frequencies and percentages for categorical variables. To examine the selection to the current study, the participant characteristics were compared between the study population included in the analyses (*n* = 551) and those participants who responded only to survey and retired also in 2014 − 2019 (*n* = 2560) using Chi squared test for categorical variables and ANOVA for continuous variables.

In the statistical analysis the proportion of time spent in each behavior was treated as compositional data. The analyses were conducted using the packages compositions [24], robCompositions [25] and nlme [26] in the statistical software RStudio (version 4.0.5). The data set included six zero values for MVPA. Because zeros cannot be included in CoDA, these values were imputed close to one minute using the R-package robCompositions.

### Descriptive analysis of compositional data

The compositional means were calculated as the component-wise geometric means of the data, and rescaled to sum up to 1440 min. To check for possible outliers, the proportions of each movement behavior before and after retirement was compared using log contrasts. Among men retiring from non-manual occupations, five individuals showed markedly different changes in the movement behaviors, that is, very large increase in the proportion of SED or LPA while the rest of the male non-manual workers’ group showed mild decreases in the proportions of both SED and LPA. Because these five individuals had unusual sleep cycle after retirement, leading to low proportions of sleep and high proportions of SED or LPA, they were considered as outliers and excluded from the analyses, resulting in a final analytical sample of 551 participants.

Ternary plots were drawn to visualize the relationships between the different sub-components of the composition using the package ggtern [27]. The plots were drawn in groups of four, one plot for each combination of three-dimensional sub-components of the four-part composition. Bootstrapped confidence regions were calculated with the package ggtern and they were based on a normality assumption and the Mahalanobis distance of the logratio transformation [28]. All the results are given by gender and occupational groups because previous studies have shown differences between these groups.

The compositional differences between pre- and post-retirement compositions were calculated for each participant via perturbation, which is a compositional operation analogous to addition or subtraction [28]. In practice, this meant scaling each observation so that the sum of its compositional parts was one [1], and then dividing each part of the post-retirement observation with the corresponding part of the pre-retirement observation. The resulting composition of the compositional differences is visualized as ternary plots.

### Compositional data analysis

An isometric logratio (ilr) transformation was used to map the compositional data into real-valued coordinates, which reduces the dimensionality of the data and allows standard statistical methods to be used [11]. The specific type of ilr coordinates used in this study were *balance coordinates.* The balance coordinates were formed by assigning compositional parts into opposing groups, with each coordinate pertaining to a positive or negative group. These groups were then used to calculate the coordinates in such a way that each coordinate represents the ratio of the sizes of its groups, in other words how much larger the combined proportional size of the parts in one group is compared to the parts in the other group [28].

The groups of compositional parts for each coordinate were chosen using sequential binary partitioning [28] (Additional file 1). First, the active behaviors, LPA and MVPA were selected as positive, and the passive behaviors, sleep and sedentary behavior were selected as negative (balance coordinate 1). These sub-compositions correspond to the first coordinate of the transformation, with positive values of the coordinate indicating that the proportion of the positive group is higher and vice versa. For the second coordinate, the parts of the previous positive sub-composition that is, active behaviors were divided, with LPA selected as positive and MVPA selected as negative (balance coordinate 2). Thus, positive values of the second coordinate corresponded to the proportion of LPA being higher in the ratio of LPA vs. MVPA. Finally, for the third coordinate, the negative sub-composition that is, the passive behaviors were divided, with positive values corresponding to the dominance of SED in the ratio of SED vs. sleep (balance coordinate 3). Compared to the first balance coordinate, the second coordinate contains information only about the relationship between LPA and MVPA and not their relationship to the other two passive components and, similarly, the third coordinate contains information only on the relationship between SED and sleep.

The binary partition created by the sequential partitioning was used to create the balance coordinate transformation. After the coordinate transformation was applied to the compositional data, a separate linear mixed model was fitted for each of the three coordinates to study the changes in the 24-h movement behaviors during the transition to retirement. In Model 1, the main effect of time on the first coordinate (active vs. passive behaviors) during the transition to retirement was examined, using gender and occupation as covariates. In Model 2, the modifying effects of gender and occupation were examined by adding interaction terms gender*time and occupation*time to the Model 1. In Model 3, the modifying effect of both gender and occupation was examined by adding the interaction term gender*occupation*time to the Model 2. All three models were repeated for the coordinate 2 (LPA vs. MVPA) and coordinate 3 (SED vs. sleep). In the models, each participant was given a random intercept and a random slope of time.

## Results

Pre-retirement characteristics of the study population are presented in Table 1. Of the study population 86% were women. Majority of women and men were non-manual workers (65% and 68%, respectively). Less than one-tenth were smokers, about half had chronic disease and mobility limitation was very rare in both women and men. There were no marked differences between the study population included in the analyses and survey-only study participants, only smoking was less prevalent among the included study participants (Additional file 2).

**Table 1:** Participant characteristics before retirement

	All	Women	Men
n (%)	551	472 (86)	79 (14)
Age, mean (SD)	63.3 (1.1)	63.3 (1.0)	63.2 (1.4)
Occupational group, n (%)			
Manual	189 (34)	164 (35)	25 (32)
Non-manual	362 (66)	308 (65)	54 (68)
Current smoking, n (%)	36 (7)	33 (7)	3 (4)
Chronic diseases, n (%)	286 (54)	246 (54)	40 (53)
Mobility limitation, n (%)	14 (3)	12 (3)	2 (3)
BMI, mean (SD), kg/m^2^	26.8 (4.6)	26.7 (4.7)	27.5 (3.8)

*BMI* Body mass index

Table 2 presents the compositional means of each 24-h movement behavior before and after the transition to retirement by gender and occupational group. Before retirement, women slept approximately 8 h per night, had 11 h SED, 4 h LPA and 50 min MVPA per day. Men had on average 16 min less sleep, one hour more SED and 46 min less LPA compared with women and the same amount of MVPA as women. Among both women and men, levels of SED tended to be lower and physical activity higher among manual workers compared with non-manual workers before retirement.

**Table 2:** The mean minutes for each 24-h movement behavior before and after retirement by gender and occupational group. The means are scaled to 1440 min (24 h)

	All	Women	Women	Women	Men	Men	Men
			non-manual	manual		non-manual	Manual
Before retirement							
Sleep	478	480	482	475	464	455	482
SED	672	663	684	622	726	749	677
LPA	239	246	228	284	200	188	228
MVPA	50	50	46	58	50	49	53
After retirement							
Sleep	515	518	517	520	492	487	504
SED	667	659	660	658	711	721	690
LPA	217	221	221	221	194	189	206
MVPA	42	42	42	41	42	43	40
Change							
Sleep	+ 36	+ 38	+ 36	+ 45	+ 29	+ 32	+ 22
SED	-6	-4	-24	+ 36	-15	-28	+ 13
LPA	-22	-25	-6	-64	-6	+ 1	-23
MVPA	-8	-9	-5	-17	-8	-5	-13

*SED* sedentary time, *LPA* light physical activity, *MVPA* moderate-to-vigorous physical activity

**Fig. 1:**
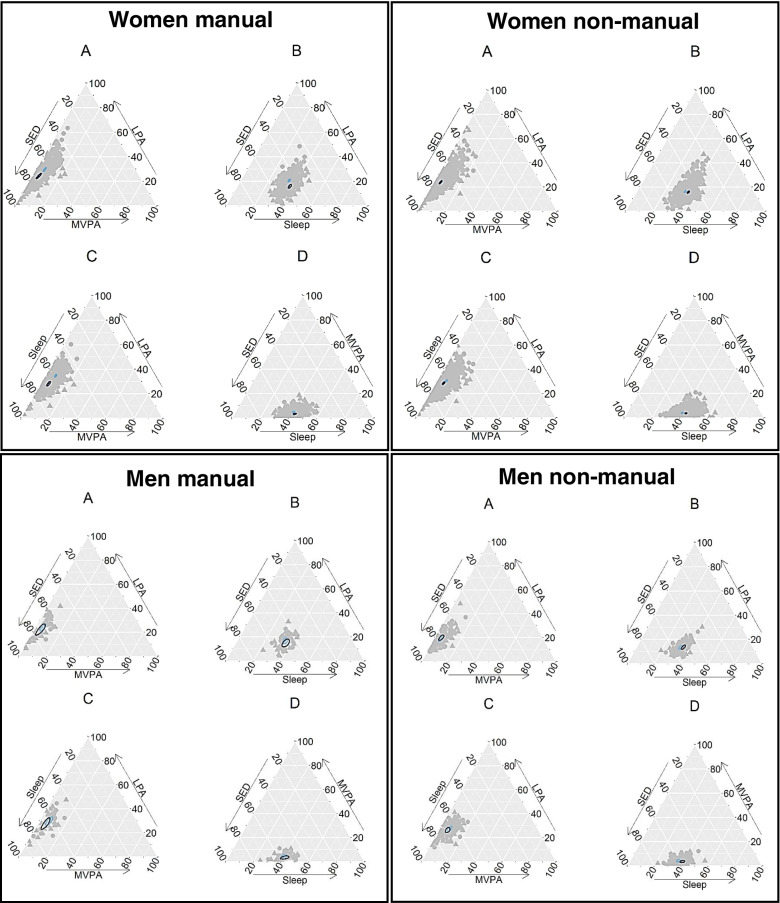
Ternary plots for all the observations before and after retirement by gender and occupational group in the three-dimensional sub-compositions (**A**, **B**, **C**, and **D**). The compositional means and their 95% confidence regions are presented for before retirement (light blue) and after retirement (dark blue). The position of a point on a plot indicates the ratio of the corresponding three components. The bootstrapped 95% confidence regions are based on an assumption of normality. MVPA = moderate-to-vigorous physical activity, LPA = light physical activity, SED = sedentary time

To illustrate changes in the compositional means during the transition to retirement by gender and occupational group, the compositional means and their 95% confidence regions were mapped to four ternary plots, one plot for each possible three-dimensional sub-composition (Fig. 1). These descriptive figures show that women retiring from manual occupations were responsible for the most marked changes in the 24-h movement behavior composition; the compositional mean shifted towards sleep and SED, implicating that both sleep and SED increased in relation to physical activity (Fig. 1, Women manual A, C). These changes were equal to on average + 45 min increase in sleep, + 36 min increase in SED, -64 min decrease in LPA and -17 min decrease in MVPA (Table 2). Among women retiring from non-manual occupations, only small differences in the compositional means were observed; slight shift towards sleep after retirement, indicating increase in sleep relative to all remaining behaviors (Fig. 1, Women non-manual B − D). Among men retiring from manual occupations, both sleep and SED increased slightly in relation to physical activity after retirement (Fig. 1, Men manual B − D), while non-manual workers tended to increase sleep in relation to SED and MVPA (Fig. 1, Men non-manual B − D).

Occupational differences in the compositional change during the transition to retirement are illustrated with ternary plots separately for women and men (Additional file 3). Among men, the observations of both occupational groups were clustered near to the center of the plot, indicating that the ratio between the three sub-components did not change markedly during the transition to retirement. Among women the observations were more scattered away from the center, indicating more notable changes. The manual group included women increasing SED and sleep markedly in relation to remaining behaviors whereas the non-manual group included some women increasing their MVPA in relation to all remaining behaviors (Additional File 3, Women A, C, D).

The compositional data were further mapped into real-valued coordinates to examine statistically with linear mixed models, how the compositional parts changed during the transition to retirement (Table 3). The ratio of active vs. passive movement behaviors (balance coordinate 1) decreased over time (*p* < 0.001), indicating that passive movement behaviors increased in relation to active behaviors during the transition to retirement. The change in the ratio of active vs. passive movement behaviors depended on occupation (time*occupation *p* < 0.001). Manual workers increased both passive behaviors, that is, sleep and SED, in relation to active behaviors after retirement. In contrast, among non-manual workers, only sleep from the passive behaviors increased in relation to both active behaviors and SED (Fig. 1 and Table 2).

**Table 3:** Estimated fixed effects of changes in the compositional parts and their 95% confidence intervals from the linear mixed models. Coordinate 1 is for active vs. passive movement behaviors, coordinate 2 for LPA (light physical activity) vs. MVPA (moderate-to-vigorous physical activity), and coordinate 3 for SED (sedentary behavior) vs. sleep

	β	95% CI		*P* value
Coordinate 1: Active vs. passive behaviors				
Model 1^a^				
Time	-0.17	-0.23	-0.12	< .001
Gender	0.11	-0.02	0.23	0.09
Occupation	0.17	0.09	0.26	< .001
Model 2^b^				
Time^*^gender	-0.06	-0.20	0.09	0.45
Time^*^occupation	-0.27	-0.38	-0.16	< .001
Model 3^c^				
Time*gender*occupation	-0.13	-0.45	0.18	0.40
Coordinate 2: LPA vs. MVPA				
Model 1^a^				
Time	0.06	0.02	0.11	0.01
Gender	0.13	0.03	0.23	0.01
Occupation	0.01	-0.06	0.09	0.75
Model 2^b^				
Time*gender	-0.04	-0.17	0.09	0.53
Time*occupation	0.01	-0.09	0.11	0.84
Model 3^c^				
Time*gender*occupation	-0.03	-0.31	0.26	0.85
Coordinate 3: SED vs. Sleep				
Model 1^a^				
Time	-0.06	-0.07	-0.04	< .001
Gender	-0.09	-0.12	-0.05	< .001
Occupation	-0.04	-0.07	-0.02	< .001
Model 2^b^				
Time*gender	-0.00	-0.04	0.03	0.89
Time*occupation	0.05	0.02	0.08	< .001
Model 3^c^				
Time*gender*occupation	-0.00	-0.08	0.08	0.92

^a^Model 1, gender and occupation as covariates

^b^Model 2, gender, occupation and gender^*^occupation term included in the model

^c^Model 3, gender, occupation, gender*occupation, gender*time and occupation*time terms included in the model

The ratio of LPA vs. MVPA (balance coordinate 2) increased over time (*p* = 0.01). The change in the ratio during the transition to retirement did not depend on gender or occupation (*p* = 0.53 and *p* = 0.84). Among all, the ratio of LPA vs. MVPA increased over time due to greater relative decrease of MVPA compared to LPA (Fig. 1 and Table 2).

The ratio between SED vs. sleep (balance coordinate 3) decreased over time (*p* < 0.001). The change during the transition to retirement did not differ between genders (*p* = 0.89), but occupational differences were observed (time*occupation *p* < 0.001). Compared with manual workers, non-manual workers’ ratio of SED vs. sleep decreased more because the proportion of sleep increased markedly in relation to all remaining behaviors, including SED, whereas among manual workers the proportions of both sleep and SED increased after retirement (Fig. 1 and Table 2).

## Discussion

This study is the first to examine how retirement changes the distribution of time spent in the 24-h movement behaviors using compositional data analysis (CoDA). Among manual workers, especially women, proportions of both sleep and SED increased in relation to physical activity after retirement. Non-manual workers’ proportion of sleep increased in relation to both physical activity and SED. Among all retirees, proportion of MVPA decreased relatively more than LPA. These findings extend previous observations regarding changes in the single component movement behaviors in the transition to retirement [3–7, 29]. The CoDA methodology allows detailed examination of changes in the 24-h movement behaviors and is applicable in studying changes during other life events or for instance during interventions.

Our findings showed that the proportion of sleep in the 24-h composition increased among all retirees, independent of gender or occupation, being the main contributor to decreasing physical activity after retirement. This is likely explained by removal of work-related activities, such as commuting, as there is no longer need to schedule sleeping times within the limits of work hours. Our previous findings have shown that increased sleep after retirement was mainly explained by delayed waking times [3], which may suggest that commuting to work has been replaced with sleeping later during mornings after retirement.

We observed that occupation modified changes in the 24-h movement behaviors during the transition to retirement. Among manual workers, not only proportion of sleep but also the proportion of SED increased. Our previous study has shown that of specific sedentary activities, especially watching TV increased after retirement [12]. Moreover, among manual workers daytime hours were more sedentary after retirement compared to workdays before retirement [5]. Thus, physical activity at work may have been partly replaced with sedentary activities such as watching TV after retirement. Among non-manual workers, increase in the proportion of sleep was the most prominent change during the retirement transition and it was accompanied by decrease in the proportions of SED and physical activity. It is possible that the proportional decrease in SED is related to removal of sedentary workhours, as our previous study has shown that non-manual workers’ daytime hours are less sedentary after retirement compared to workdays before retirement [5].

We used occupational status as an indicator of work-related activities but occupation is also an indicator of socioeconomic status (SES) [30]. Worktime as well as leisure time physical and sedentary activities seem to differ between SES groups as lower SES groups are active at work but sedentary during leisure time, while higher SES groups are more sedentary at work but more active during leisure time than lower SES groups [30–32]. This may also be reflected to changes in movement behaviors after retirement, given that work activities are replaced by leisure activities. Future studies with more detailed information on SES based not only on occupation but also on education and income are needed to elaborate the role of SES in the changes in movement behaviors during the transition to retirement.

The results of the current study are in line with our previous findings, indicating lower SED levels and higher total physical activity levels among women compared with men both before and after retirement [5, 7]. Possible explanations for the gender differences are that household chores and active commuting are more common among women compared to men in this age group [33, 34] and there are also gender differences in occupations [35]. However, no marked differences were observed in changes in the 24-h movement behaviors across the retirement transition between women and men. It should be noted that low number of men in the sample decreases the robustness of the findings on gender differences. Future studies with larger and more gender balanced samples should be conducted to confirm the findings.

Decrease in the proportion of physical activity after retirement was relatively more visible in MVPA than LPA independently from gender and occupation, suggesting decreased intensity of physical activity after retirement. One explanation may be removal of active commuting that is a relatively common activity in our study population, as approximately 20% of women and 14% of men reported commuting by walking or cycling [7]. Our previous work has shown that actively conducted trips, such as commuting on workdays contribute markedly to daily MVPA in this age group [36]. In general, aging seems to be related to decreasing intensity of physical activity [37], but it is unlikely the explanation for our findings, given that the follow-up time was only one year and the study population was relatively active and healthy.

Regarding the clinical relevance of the findings, a recent federated analysis from cohort studies showed that lower SED and higher physical activity (of any intensity) are associated with lower all-cause mortality [38]. There is also some evidence from cross-sectional studies that replacing MVPA with any other movement behavior (sleep, SED, LPA) is associated with increase in adiposity and deterioration of other cardiometabolic risk markers [8]. These studies imply that our finding of replacing active behaviors with passive behaviors within a 24-h day after retirement may have unfavorable health consequences. Overall decreases of MVPA seemed to be relatively high, up to 17 min per day. On the other hand, non-manual workers seemed to replace some SED with sleep, which may benefit health for those with inadequate sleep before retirement [8]. Given that retirement concerns removal of work-related physical activity, and leisure vs. work-related physical activity may have different health implications [39, 40], future studies should examine the health effects of changes in the 24-h movement behaviors across the transition to retirement.

Our present observations give some prospects for practice and future interventions targeted to retiring workers. Overall, the results of the current study imply that more attention should be paid to physical activity during and after the retirement transition. There is need to develop and evaluate strategies to maintain or increase physical activity and decrease sedentary behavior in this population group. We found that increases in sleep and SED affected relatively more MVPA than LPA, suggesting that promotion of especially MVPA is needed. However, for the most sedentary retirees, more practical approach may be to start reducing SED by increasing LPA [41].

The main strengths of the current study are the longitudinal study design, annual accelerometer measurements covering the entire 24-h movement behavior composition before and after retirement, and the application of CoDA which enabled examinations of how retirement changes the distribution of time spent in the 24-h movement behaviors. Moreover, the study population included a variety of occupations, which enabled us to examine changes in the 24-h movement behavior composition separately among manual and non-manual workers. Finally, health-related selection to early retirement did not bias our study as all participants retired on a statutory basis.

Naturally, our study also has some limitations. The threshold values used to identify SED, LPA and MVPA were developed in small study populations of younger adults, among 20-year-olds (*n* = 20) [20] and 30-year-olds (*n* = 30) [19]. However, we are not aware of threshold values developed among the similar study population with ours (healthy 65-year olds with no marked mobility limitations). Moreover, the absolute estimates of sedentary time from wrist-worn accelerometers may be different from estimates from the more accurate posture-based methods [42]. In our study population, physical activity levels estimated by a wrist-worn accelerometer were relatively high (50–60 min of MVPA per day). However, similar levels have been reported in another wrist-accelerometer-based study among older adults applying similar data processing methods [43]. Possible inaccuracy in the absolute values of 24-h movement behaviors should not influence the interpretation of the findings, because we focused on relative, intra-individual changes in the whole 24-h movement behavior composition rather than absolute values of each 24-h movement behavior. Our findings concern relatively short-term one-year changes in movement behaviors, thus future studies should examine how movement behaviors change in the long-term across the transition to retirement.

Regarding generalizability, there were no marked differences between the current study population and the eligible study population that is, Finnish public sector employees transitioning to statutory retirement. The number of men in the sample was small, which limits the generalizability of the findings to men. However, this is representative of the eligible study population of whom 78% are women [44]. However, noteworthy is that the participants entered to statutory age-based retirement, and therefore are generally healthier compared with those who retire early due to for instance disability or unemployment. Moreover, results are not necessarily generalizable to other countries with different pension age and pension system, and should be confirmed in future studies with different study populations.

## Conclusions

Retirement induced a decrease in the proportion of time spent in active behaviors, especially time spent in MVPA. Among manual workers proportions of both sleep and SED contributed to decreasing proportion of activity, while non-manual workers’ proportion of sleep increased in relation to both SED and active behaviors. Future studies are needed to find ways to maintain or increase daily physical activity levels at the cost of sedentary behaviors among retirees.

## Supplementary Information

**Additional file 1.** The sequential binary partition used for the balance coordinate transformation.

**Additional file 2. **Comparison of the characteristics between the study population included in the analyses (*n*=551) and the survey-only study population (*n*=2560) in the last available measurement in which the participantswere still working.

**Additional file 3. **Ternary plots for the compositional differences between before and after retirement by gender and occupational group.

## Data Availability

Anonymised partial datasets of the FIREA study are available by application with bona fide researchers with an established scientific record and bona fide organisations. In case of data requests, please contact the principal investigator Sari Stenholm, sari.stenholm@utu.fi.

## Abbreviations

BMI: Body mass index
CI: Confidence interval
CoDA: Compositional data analysis
FIREA: The Finnish Retirement and Aging Study
ISCO: The International Standard Classification of Occupations
LPA: Light physical activity
MET: Metabolic equivalent
MVPA: Moderate-to-vigorous physical activity
SD: Standard deviation
SED: Sedentary behavior
