# Supplementary material for: Changes in the 24-h movement behaviors during the transition to retirement: compositional data analysis
Source: Int J Behav Nutr Phys Act. 2022 Sep 15;19:121. doi: 10.1186/s12966-022-01364-3 (PMC9479436; doi:10.1186/s12966-022-01364-3)
Supplement: Supplementary file 1 — Additional file 1. The sequential binary partition used for the balance coordinate transformation. [file 12966_2022_1364_MOESM1_ESM.docx]

**Additional file 1.** The sequential binary partition used for the balance coordinate transformation. Each column indicates the parts of the group for each coordinate. The value -1 indicates that the part belongs to the negative group, the value 1 indicates that the part belongs to the positive group, and the value 0 indicates that the part is not included to the coordinate. SED=sedentary time, LPA=light physical activity, MVPA=moderate-to-vigorous physical activity.

| **Coordinate** | **1** | **2** | **3** |
| --- | --- | --- | --- |
| SED | -1 | 0 | 1 |
| LPA | 1 | 1 | 0 |
| MVPA | 1 | -1 | 0 |
| Sleep | -1 | 0 | -1 |
