# Supplementary material for: Changes in the 24-h movement behaviors during the transition to retirement: compositional data analysis
Source: Int J Behav Nutr Phys Act. 2022 Sep 15;19:121. doi: 10.1186/s12966-022-01364-3 (PMC9479436; doi:10.1186/s12966-022-01364-3)
Supplement: Supplementary file 2 — Additional file 2. Comparison of the characteristics between the study population included in the analyses (n=551) and the survey-only study population (n=2560) in the last available measurement in which the participantswere still working. [file 12966_2022_1364_MOESM2_ESM.docx]

**Additional file 2.** Comparison of the characteristics between the study population included in the analyses (n=551) and the survey-only study population (n=2560) in the last available measurement in which the participants were still working.

|  | Study population  n=551 | Survey-only study population n=2560 | P value |
| --- | --- | --- | --- |
| Age, mean (SD) | 63.3 (1.1) | 63.4 (1.5) | 0.10 |
| Women, n (%) | 472 (86) | 2117 (83) | 0.09 |
| Occupational group, n (%) |  |  | 0.36 |
| Manual | 189 (34) | 923 (36) |  |
| Non-manual | 362 (66) | 1615 (64) |  |
| Current smoking, n (%) | 36 (7) | 247 (10) | 0.03 |
| Chronic diseases, n (%) | 286 (54) | 1290 (51) | 0.22 |
| Mobility limitation, n (%) | 15 (3) | 85 (3) | 0.50 |
| BMI, mean (SD), kg/m^2^ | 26.8 (4.6) | 26.9 (4.4) | 0.80 |
| Self-reported leisure time physical activity, MET-hours/week, mean (SD) | 24.2 (18.5) | 23.0 (19.6) | 0.17 |

BMI=Body mass index
