# Supplementary material for: Changes in the 24-h movement behaviors during the transition to retirement: compositional data analysis
Source: Int J Behav Nutr Phys Act. 2022 Sep 15;19:121. doi: 10.1186/s12966-022-01364-3 (PMC9479436; doi:10.1186/s12966-022-01364-3)

**Additional file 3.** Ternary plots for the compositional differences between before and after retirement and their 95% confidence regions by gender for manual workers (dark purple dots and line) and non-manual workers (light purple dots and line) in the three-dimensional sub-compositions (A, B, C, and D). The ratio between the components in each plot indicates the change in the proportions during the transition to retirement. Observations clustered near to the center of the plot indicate that the ratio between the three sub-components did not markedly change over time. The 95% confidence regions are based on an assumption of normality. MVPA=moderate-to-vigorous physical activity, LPA=light physical activity, SED=sedentary time.


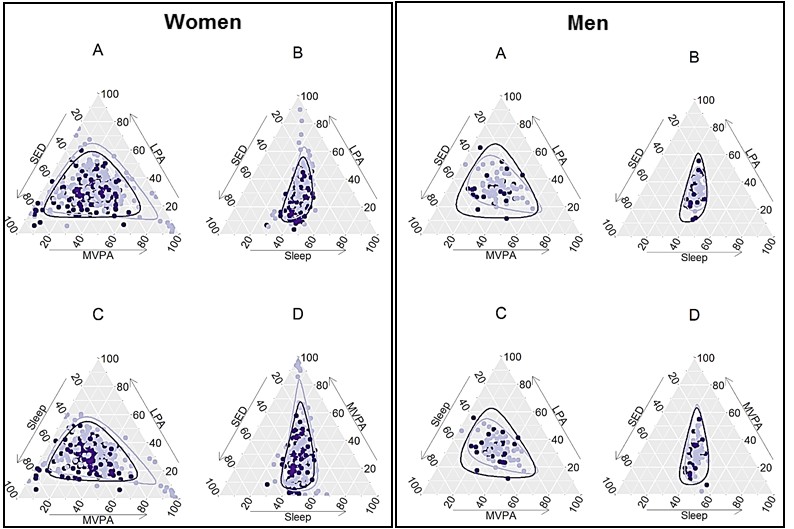

Supplement: Supplementary file 3 — Additional file 3. Ternary plots for the compositional differences between before and after retirement by gender and occupational group. [file 12966_2022_1364_MOESM3_ESM.docx]
